# Photographing the Unseen

**Published:** 1896-05

**Authors:** Howard Van Rensselaer

**Affiliations:** Associate Professor of Materia Medica, and Lecturer on General Medicine in the Albany Medical College.


AMERICAN JOURNAL

—OF—

DENTAL SCIENCE.

VOL. XXX. BALTIMORE, MAY, 1896. NO. 1.

ARTICLE I.

### PHOTOGRAPHING THE UNSEEN.

BY HOWARD VAN RENSSELAER, PH. B., M. D.,

Associate Professor of Materia Medica, and Lecturer on General Medicine in the Albany Medical College.

When the discovery was first made that images of visible objects could be caught and fixed upon a sensitized plate, the process being called photography, it probably did not excite a tenth of the interest and amazement among scientific men that followed the announcement of Professor William Conrad Roentgen of Wurzburg university, Austria, a few weeks ago, that he had discovered that it was possible for certain rays to penetrate many opaque objects; and that the image of the unseen could be developed and shown upon an ordinary photographic plate.

Wonderful, indeed, it seems that transparent substances like clear glass are opaque to these rays, while dense materials like flesh, paper and wood, permit the rays to pass more or less readily through them.

Almost at the very beginning investigators found that these rays did not obey the rules which govern white light, in that they could not be focused or dispersed, nor could they be reflected nor refracted by ordinary methods; nor were they capable of deviation by strong magnets, as are the cathode rays, but that they are entirely anomalous and peculiar to themselves.

The word photography in connection with them is also a misnomer, as the rays themselves are invisible and cannot be considered light in any ordinary acceptation of the term, and instead of a true picture of the object, its shadow simply is thrown upon the plate.

The course of the rays, called by the discoverer X rays, was traced by means of a fluorescent screen, and their penetration of opaque objects demonstrated by plac ing organic bodies before this screen.

Professor Roentgen's original investigation was made with a Crooke's tube, which is an irregularly shaped vessel or tube of thin glass, through the walls of which are hermetically sealed electrodes of platinum; the tubes are then exhausted of air, to less than a millionth of an atmosphere, affording the nearest approach to a perfect vacuum that has ever been produced.

Through this tube an electric current from a powerful Ruhmkorff coil is passed, which develops at the cathode or negative pole a faint, bluish light, which throws upon the glass surface a brilliant phosphorescence. The rays of light from the cathode to the walls of the glass are called the cathode rays.

The so-called X rays, as they are named by the discoverer, which are invisible, appear to proceed from the phosphorescence on the inner walls of the tube, or better still, through an aluminum window let into the tube.

These X rays, while they resemble the cathode rays in many respects, are not identical with them, as the latter are visible and can be deviated by a magnet, while the former are invisible and cannot be turned aside from their course by any known agent. When a screen which may exhibit phosphorescence is interposed in front of X rays, its surface will appear luminous.

In his first experiment, the Crooke's tube was enveloped in black paper, and then the florescent screen was placed near it; the screen became at once luminous, showing that the invisible rays had penetrated the black paper. A book of a thousand pages was then interposed between the tube and the screen, but the luminosity continued; a block of wood, and a sheet of aluminum were experimented on with like result; a sheet of iron, however, intercepted the rays, showing that certain substances only permitted these rays to pass, while others were opaque to it.

When the living hand was placed in front of the screen a very curious result was observed. It was found a shadow of its osseous skeleton was obtained on the luminous surface, and that the image of a ring that was on one of the fingers was particularly sharp and distinct.

The next experiment was to ascertain what effect, if any, these newly discovered rays would produce upon a photographer's plate. That a shadow would be impressed upon the sensidized film, which could be developed by the ordinary methods, he already knew; as some time ago Hertz had demonstrated that these rays, though passing through glass with difficulty, would readily permeate a window of aluminum if inserted in the tube in place of the glass, and would excite phosphorescence on a suitable screen, and chemical action on certain salts; and again, Lenard, two years ago, by means of the Crooke's tube, had obtained shadowgraphs on photographic plates.

The feature that was peculiar to Roentgen's experiments, and the only fact new to science was, that bony structures are impenetrable to these rays, while flesh is nearly transparent.

It was this startling and sensational fact that a living person,s bones could be photographed, that stirred the public mind, and aroused so wide spread an interest among the members of the medical profession. The public papers, scientific magazines and especially medical journals have since teemed with the results obtained by many experimenters. In a general way the results so far to the medical profession are as follows :

That in the thinner portions of the body, as the hands, arms and feet, it is possible to establish the shape and location of foreign bodies, as bullets, metals and glass that lie buried in the soft tissues and concealed from the eye, provided that they are not covered by the bony structures; that fractures, dislocations, exostoses and some pathological alterations of the bones can also be recognized.

This is as far as our knowledge has advanced as yet. That we shall ever be able to view lesions or even foreign bodies within the brain seems improbable, on account of the opacity of the bones of the cranium which obstructs these rays.

All the soft tissues so far examined seem equally translucent, and no attempt to demonstrate pathological alterations of the soft tissues or organs, within the body or in the laboratory, have been successful.

So, as yet, its application to the medical profession is strictly limited within narrow bounds; but there has been opened a limited field for research and experiment in many directions. Perhaps special plates prepared with fluorescent or other substances may be discovered that will lessen the time of exposure, which is now from many minutes to two hours, and may perhaps bring out details in the picture which now are lost in the uniform darkness of a silhouette.

The non-refractability of the rays prevent our focussing them, so that many of the outlines are still indistinct, and it is impossible to make a reduced image, the shadow being practically of the same size as the object. Possibly these objections may be overcome.

Undoubtedly in the near future physicians will invent improved, cheaper and simpler apparatus for more accurate scientific research. The physiologist, it is hoped, may then elucidate ways of measuring the gradations of the permeability of the rays through all animal tissues. When this occurs the clinician may be able to use these methods for more accurate diagnosis; and it is even within the bounds of possibility that the pathologist may employ these rays to modify the action of diseased tissues; though at the present the few experiments that have been made in this direction are not encouraging, as bacteria, which have had prolonged exposure to the action of these rays, are uninfluenced by them; and the living human eye, when interposed in the direct path of the X rays, is not cognizant of any effect of light, or of heat, or of pain, or, in fact, of any sensation.

Already many prophecies have been made of what we may hope and reasonably expect from these newly discovered rays, but they are at present but mere speculation, and, from the point of pure science, we are still on the threshold of the great unknown, and it is impossible now to predict what the result will be.—*Albany Medical Annals.*

